# Deep Learning of Histopathological Features for the Prediction of Tumour Molecular Genetics

**DOI:** 10.3390/diagnostics11081406

**Published:** 2021-08-03

**Authors:** Pierre Murchan, Cathal Ó’Brien, Shane O’Connell, Ciara S. McNevin, Anne-Marie Baird, Orla Sheils, Pilib Ó Broin, Stephen P. Finn

**Affiliations:** 1 Department of Histopathology and Morbid Anatomy, Trinity Translational Medicine Institute, Trinity College Dublin, D08 W9RT Dublin, Ireland; murchanp@tcd.ie (P.M.); obriec12@tcd.ie (C.Ó.); MCNEVINC@tcd.ie (C.S.M.); 2 Department of Histopathology, St James’s Hospital, P.O. Box 580, James’s Street, D08 X4RX Dublin, Ireland; 3 School of Mathematics, Statistics, and Applied Mathematics, National University of Ireland Galway, H91 TK33 Galway, Ireland; s.oconnell29@nuigalway.ie (S.O.); pilib.obroin@nuigalway.ie (P.Ó.B.); 4 Department of Medical Oncology, St James’s Hospital, D08 NHY1 Dublin, Ireland; 5 School of Medicine, Trinity Translational Medicine Institute, Trinity College Dublin, D02 A440 Dublin, Ireland; bairda@tcd.ie (A.-M.B.); osheils@tcd.ie (O.S.)

**Keywords:** histopathology, deep learning, cancer, molecular diagnostics

## Abstract

Advanced diagnostics are enabling cancer treatments to become increasingly tailored to the individual through developments in immunotherapies and targeted therapies. However, long turnaround times and high costs of molecular testing hinder the widespread implementation of targeted cancer treatments. Meanwhile, gold-standard histopathological assessment carried out by a trained pathologist is widely regarded as routine and mandatory in most cancers. Recently, methods have been developed to mine hidden information from histopathological slides using deep learning applied to scanned and digitized slides; deep learning comprises a collection of computational methods which learn patterns in data in order to make predictions. Such methods have been reported to be successful in a variety of cancers for predicting the presence of biomarkers such as driver mutations, tumour mutational burden, and microsatellite instability. This information could prove valuable to pathologists and oncologists in clinical decision making for cancer treatment and triage for in-depth sequencing. In addition to identifying molecular features, deep learning has been applied to predict prognosis and treatment response in certain cancers. Despite reported successes, many challenges remain before the clinical implementation of such diagnostic strategies in the clinical setting is possible. This review aims to outline recent developments in the field of deep learning for predicting molecular genetics from histopathological slides, as well as to highlight limitations and pitfalls of working with histopathology slides in deep learning.

## 1. Introduction

A large number of cancer treatments such as chemotherapies and radiotherapies are characterised by adverse side-effects, largely due to targeting both cancerous and healthy cells. Recent research into novel treatment strategies such as immunotherapies and targeted therapies aims to reduce severe side-effects while improving treatment response and quality of life. Immunotherapeutic drugs are designed to stimulate or suppress the patient’s immune response, while targeted therapies aim to interrupt specific genes or proteins which drive cancer development. Successfully applying these precision cancer treatments is often dependent on tumour-specific molecular features. Therefore, accurate biomarker testing is crucial in identifying patients with a favourable response to treatment. For example, in lung cancer, the genotype of epidermal growth factor receptor (EGFR) guides the use of treatment with multiple tyrosine-kinase inhibitors targeted to the mutated EGFR protein [1]. In melanoma, mutated BRAF is directly targetable with the drug vemurafenib [2], among others. These predictive oncogenic mutations are typically referred to as clinically actionable mutations. Gene expression is also known to play an important role as a predictive biomarker in certain treatments. For example, non-small-cell lung cancer (NSCLC) patients with high TP53 expression have been found to have greater survival benefit from adjuvant cisplatin [3], while PD-L1 protein expression has demonstrated utility in patient selection for treatment with immune checkpoint inhibitors [4]. However, PD-L1 expression has been criticised as an imperfect predictive biomarker due to intratumoural and intertumoural heterogeneity [5], leading to the investigation of numerous other biomarkers including tumour mutational burden (TMB) and microsatellite instability (MSI) as surrogate biomarkers for tumour antigenicity and/or PD-L1 expression [6,7].

Patient stratification for treatment is critical given the significant cost and risk associated with modern cancer therapies. Such therapies aim to improve patient survival rates while also enabling an acceptable quality of life during and after treatment. Despite the advantages of precision oncology, limitations in molecular assays remain an obstacle to their wide-spread adoption. Molecular tests rely on obtaining tissue samples with high tumour purity, and often come at high cost and turnaround time. In contrast, histopathological assessment of tumour slides is considered routine, and in many cases mandatory, in cancer diagnosis. It has been shown that molecular biomarkers are sometimes associated with morphological alterations in certain cases, for example, BRAF mutations in melanoma cases have been associated with larger, rounder and more pigmented tumour cells [8], while EGFR-mutant lung adenocarcinomas have been found to be characterised by hobnail cell types [9]. Such histopathological biomarkers, assessed by a trained pathologist, could prove critical in designing treatment strategies as well as determining tumour subtypes and predicting prognosis. However, inter-pathologist variation, the need for objective and quantitative assessments in diagnosing cancer cases, as well as staff shortages in histopathology departments and increased workloads, motivates research into new diagnostic strategies [10,11].

Deep learning is well established in image classification and has seen a number of successful use-cases in medical imaging, both in a research and real-world setting [12]. While advances in computing capabilities have substantially contributed to the escalating potential of deep learning, the role of massive data curation initiatives, often undertaken by collaborations between researchers, cannot be overstated. Notably, the Imagenet database, first presented in 2009, has provided researchers around the world with the capability to train deeper, and more sophisticated, neural networks, in less time and with limited computing power, through what has become known as transfer learning [13]. Transfer leaning typically involves pre-training a state-of-the-art neural network on a specific problem before applying the trained network as a starting point when training for a new domain. Despite decreasing costs of computing power available through cloud providers, transfer learning remains common practice in many deep learning applications, particularly when curating massive data sets is impractical or costly.

### 1.1. Deep Learning

Machine learning can be described as a set of computational algorithms that can detect and leverage patterns in data for predictive applications. Machine learning approaches can be broadly categorised as supervised or unsupervised. In supervised learning, models are trained using ground truth labels, whereby the distance of the prediction from this label, represented by an objective cost function, is used to update the weights that describe the inputs’ relationships with the output. Examples of supervised machine learning algorithms include support vector machines, random forest classifiers, and elastic net regression models. Alternatively, unsupervised learning algorithms attempt to detect patterns in data without using ground truth data labels, and often operate via a set of rules that describe similarity between individual instances. Therefore, a common application of unsupervised learning is clustering, in which a model groups instances into discrete categories based on their similarity to each other. In the case of imprecise data labelling, a third approach termed multiple-instance learning (MIL) may also be implemented. A MIL scheme is arranged such that collections of data examples, termed bags, constitute a number of unlabelled instances and there is one label per bag. The objective of a MIL model is to predict the label of unseen bags.

Unlike traditional machine learning approaches, deep learning models differ in their architectural details. Deep learning models are comprised of an arbitrary number of layers of list based vectors, or neurons, which connect inputs to subsequent layers sequentially via weights vectors. In fully-connected architectures, the value of a particular neuron at a given layer is determined by the sum-weighted multiplication of all values at the previous layer by their respective weights. These sequential sum-weighted multiplications can transform inputs in a non-linear fashion, as each subsequent layer will be the sum-weighted combination of iterative transformed data representations. Each layer can be thresholded by an activation function, such as the rectified linear unit, to further transform the output passed to subsequent layers. For supervised applications, the final layer output is evaluated by a loss function that describes the distance from the ground truth label to the prediction. This loss quantity can then be used to update all weights in the model via backpropagation, which makes use of the gradient of the output with respect to the weights at each layer. This backpropagation procedure is carried out iteratively until the loss function is minimised and predictive performance is maximised. A more detailed discussion of backpropagation can be found in LeCun et al. [14]. These aforementioned non-linearities allow deep learning models and neural networks to capture abstract mathematical relationships between input features and labels. The neural network architecture refers to the organisation of layers and hidden units and plays an important role in the performance and computational cost of deep learning models. A number of other subjective neural network hyperparameters exist, which, unlike the weights vectors, are not learned directly from the data and must be initially chosen by the researcher, such as activation function, learning rate and batch size. These factors can also impact model performance and can be optimised during the course of training [15].

In order to assess model performance, data sets are commonly partitioned into training, validation and testing sets before model training. To further validate claims of model performance, external validation on separate data sets is often recommended. While the training data set is used to initially fit the deep learning model, the validation data set is used to obtain an unbiased evaluation of model performance during hyperparameter optimisation. The final model fit is then evaluated on a held-out test set and the performance of the model on this data set is usually the final reported quantity.

#### 1.1.1. Convolutional Neural Networks

Convolutional neural networks (CNN) are a specific class of neural network most commonly applied to imaging data, in which the general framework is similar to the previously described procedure. CNNs apply convolutional operations by sliding a filter of a specified size across an input feature and performing matrix multiplication at each location resulting in an output feature map. These feature maps mean that weights (per map) are shared across the entire image, meaning that individual feature maps, with their own convolutional weights vectors, can recognise data patterns regardless of their position in the image. This confers a useful spatial invariance property. In addition to convolutional layers, CNNs are also composed of pooling layers which reduce the dimensionality of features. Consequently, low-level features, such as edges and basic shapes, detected by early layers in the neural network can be aggregated into higher-level image features. CNNs usually flatten the transformed image features into a list based vector which can then be passed into a standard neural network architecture without spatial invariance as described previously to obtain a final prediction output [16].

#### 1.1.2. Generative Adversarial Networks

Generative adversarial networks (GAN) are a class of deep learning algorithm which implements two competing neural networks. One neural network, termed the generator, produces synthetic examples of the input data while the second neural network, termed the discriminator, attempts to evaluate whether or not a given example was generated by the generator. The objective of the GAN is to generate synthetic examples of data which appear to be drawn from the same distribution as the input data. This is achieved by minimising the classification error of the discriminator, forcing the generator to learn the structure of the input data.

### 1.2. Deep Learning Workflow in Digital Pathology

**Figure 1:**
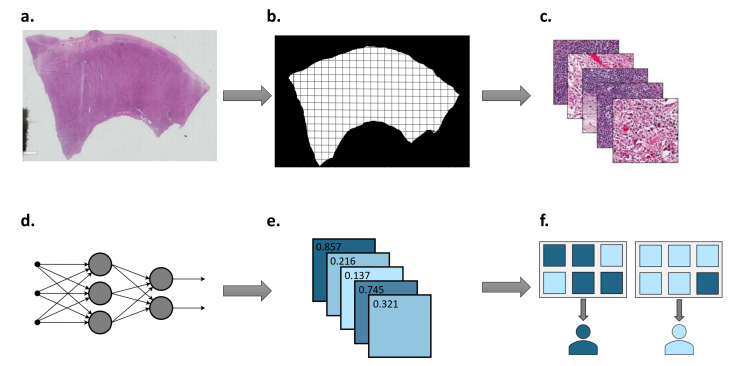
Summary of deep learning workflow in digital pathology. (**a**) Histopathology slides are scanned at various magnifications to generate WSI. (**b**) Tumour region is located and divided into tiles. (**c**) WSI tiles are colour normalised and desired augmentations are carried out. (**d**) Neural network is trained using tiles and corresponding labels. (**e**) Tile-level predictions are returned by the neural network. (**f**) Patient-level predictions are determined by aggregating tile-level predictions.

Deep learning has seen numerous successes in digital pathology, in applications ranging from tumour grading in prostate cancer [17] to survival prediction in colorectal cancer [18]. In recent years, researchers have increased their focus on using deep learning to recognise morphological patterns in histopathology images associated with molecular genetics. Given the routine availability of histopathology slides in cancer cases, this approach promises both cost- and time-effective solutions to treatment selection for cancer patients. However, considering the high dimensionality of whole-slide images (WSIs) and non-standardised approaches to sample preparation, systematic pre-processing of data is required. A summary of a typical deep learning workflow in digital pathology is shown in Figure 1.

#### 1.2.1. Sample Preparation and Annotation

Routine histopathological assessment of tumour tissue is typically undertaken on formalin-fixed paraffin-embedded (FFPE) tissue specimens. FFPE preservation has several advantages over frozen tissue samples, such as superior conservation of cellular morphology and less costly storage [19]. However, FFPE preservation can result in cross-linking, degradation, and fragmentation of DNA, making it less preferable than frozen alternatives for molecular testing [19]. Both FFPE and frozen specimens have been used in deep learning applications to histopathology; however, greater availability of FFPE samples and their suitability to long-term storage may increase their popularity with deep learning researchers. Following slide preparation and staining, histopathology slides are typically scanned at various magnifications using brightfield illumination, resulting in giga-pixel size WSIs. In order to overcome the high dimensionality of WSIs, small square regions, often referred to as tiles or patches, are extracted and tiles containing a specified proportion of background pixels are removed from the data set. Gold-standard annotation of data remains a major challenge for deep learning training data sets, and is often carried out manually. For example, the 14 million images contained in the Imagenet database were annotated through crowd-sourcing; however, in domains such as medical imaging, annotation must be carried out by trained professionals, often increasing the cost of such projects. Furthermore, WSIs typically contain both tumour and normal tissue, which may reduce the performance of deep learning models. Consequently, tumour tissue is often delineated by a trained pathologist in order to achieve tile-level annotation. The high cost and time associated with WSI segmentation has motivated research into deep learning methods to obtain automatic pixel-level annotations from slide-level labels. It has been shown that ensemble segmentation models composed of multiple fully convolutional architectures achieve superior segmentation performance compared to models composed of a single neural network [20], while other deep learning based segmentation methods have already seen use as decision support tools for pathologists [21]. In addition to tumour/normal segmentation, in deep learning applications to predicting molecular genetics, molecular assays must be undertaken on large data sets to ascertain the correct labels required for training.

#### 1.2.2. Colour Normalisation and Augmentation

The requirement of large data sets for training deep learning models in digital pathology often results in WSIs collected across multiple research centres being integrated into a single data set. However, variations in WSI preparation may result in batch effects across images which must be mitigated to reduce bias and improve the generalisability of models [22]. For example, varying concentrations and volumes of stain used in slide preparation, as well as exposure to light during storage, may result in biases between WSIs. Additionally, inter-scanner variability may further exacerbate such biases. Recent research also suggests that WSIs preserve site-specific information which can be learned by a deep learning algorithm, resulting in overestimation of model performance [23]. Stain colour normalisation aims to mitigate such batch effects by transforming pixel values from different WSIs within a data set to a common distribution. On the other hand, colour augmentation attempts to improve a model’s ability to generalise to unseen data by simulating realistic colour variations. Greater validation accuracies have been reported after applying normalisation and augmentation to WSIs [24].

#### 1.2.3. Transfer Learning and Tile Aggregation

Around the same time that the Imagenet database was released, the annual competition known as the ImageNet Large Scale Visual Recognition Challenge was formed [25]. This challenge encouraged teams of researchers from all over the world to push the boundaries of deep learning models by developing more sophisticated and efficient models, resulting in a number of architectures which have been re-purposed to a diverse range of imaging problems, including digital pathology. In addition to repurposing robust model architectures, researchers can make use of existing model parameters that were trained on data from a different dataset or domain, such as the Imagenet data set, to initialise other models and speed up training processes. These existing model parameters can be frozen, meaning that they will not be updated during backpropagation. This method, termed transfer learning, is a flexible approach to reducing compute time and making use of weight space solutions that have already shown promise in other pattern recognition tasks. For example, all ImageNet layers except the final output layer may form the basis of a predictive model in a different domain and undergo freezing, with an additional randomly initialised softmax layer providing the output for the target domain. This allows the training process to tune the weights of just one layer as opposed to a large and arbitrary number of layers. This has the useful property of mitigating sample size requirements in the target domain by using existing model parameters trained on larger data sets. Such practices are common in digital pathology due to the high cost and time involved in curating large training data sets. A more detailed discussion of transfer learning can be found in Tan et al. [26]. Deep learning models in digital pathology return predictions at the tile-level; therefore, it is necessary to implement a strategy of aggregating per-tile predictions in order to obtain a per-slide prediction. Methods of tile aggregation range from majority voting to MIL approaches [27,28].

#### 1.2.4. Model Interpretation

Lack of interpretability remains a major obstacle to the widespread adoption of deep learning systems in healthcare [22]. Meaningful understanding of deep learning predictions is crucial in healthcare in order to instil trust from both a clinician and patent’s perspective, and hence enable clinical translation. Deep learning models are often scrutinised to remove the "black-box" label typically associated with such methods, and mitigate the risk of artefacts in the data being exploited by models to make predictions. Various methods have been developed to dissect the predictions of deep learning models into human-interpretable features, such as class activation mapping [29], layer-wise relevance propagation [30] and saliency maps [31]. These methods help identify the degree to which regions of input images contribute to a model’s prediction, however, such regions may not necessarily correspond to human-interpretable features.

## 2. Materials and Methods

The search was carried out on the PubMed and Scopus Web of Science electronic databases, and included studies retrieved as secondary documents by Scopus Web of Science. The search strategy combined terms referring to “histopathology/whole slide image”, “deep learning/neural network”, “cancer/tumour” and “mutation/genetic alteration/burden/TMB/expression/microsatellite instability”. Only publications released between 1 January 2018 and 30 April 2021 were considered for selection. Following the removal of duplicates, abstracts were retrieved and reviewed for relevance using the following inclusion criteria:

1. Full-text available in English.
2. Not a review article, commentary or editorial.
3. Digitised whole-slide images are used as an input to a neural network.
4. The neural network is used to predict the presence of a molecular feature, namely, mutations, mutated genes, gene expression, hormone receptor status, TMB, or MSI.

The described search strategy resulted in a total of 31 studies which met the inclusion criteria. These studies were classed into four categories based on the type of molecular feature, which was predicted. Namely, studies were categorised as either predicting (1) mutations or mutated genes, (2) gene expression or hormone receptor status, (3) TMB, or (4) MSI.

## 3. Results

### 3.1. Predicting Mutations

The landscape of somatic mutations across different types of cancer can vary vastly, however, cancers of the same type are often characterised by specific mutations. For example, up to half of melanoma cases have been found to harbour activating mutations at BRAF V600 [32], while EGFR mutations have been found to be present in over 30% of NSCLCs [33]. As previously described, the genotype of specific genes in cancers can play a large role in influencing the course of treatment for patients. Identification of these clinically actionable mutations typically involves gene panel testing, in which specific genomic locations are assayed. However, such comprehensive genetic tests require relatively large amounts of tissue with high tumour purity and often come at significant cost. Therefore, researchers have focused on predicting genetic alterations from readily available haematoxylin and eosin (H&E)-stained WSIs using deep learning. This section summarises recent literature concerned with identifying mutations and mutated genes in cancer using deep learning applied to histopathology slides. Identified studies concerned with predicting mutations from WSIs using deep learning are summarised in Table 1.

The first peer-reviewed study which attempted to predict the mutation status of selected genes using deep learning applied to WSIs was published by Coudray et al. in 2018 [34]. In this study, the genotype of ten commonly mutated genes in lung adenocarcinoma was estimated using an Inception-v3 neural network, six of which could be predicted with AUCs greater than 0.7. Interestingly, the authors found that random classification was achieved by the model when using images tiled at 5× magnification, while accurate genotype predictions could be made at 20× magnification. Furthermore, while the deep learning model was trained using scanned frozen tissue slides, a reasonable level of generalisability was observed when applying the model to predict EGFR status in FFPE lung adenocarcinoma slides. Multiple studies have extended these findings to other cancer types using a range of methods.

For example, Chen et al. predicted the mutation status of four genes from H&E histopathology slides in liver cancer [35], while Yang et al. predicted the presence of mutations in immune-related genes in lung cancer [36]. Wu et al. applied a unique approach of integrating a tumour classification score derived by one neural network and a mutation classification score derived by a second neural network to generate a final prediction regarding the mutation status of a given tile [37]. This approach was trained using papillary thyroid carcinoma cases to predict BRAF V600E mutation status, achieving a reported AUC of 0.88. In order to better facilitate the identification of muscle-invasive bladder cancer patients with favourable outcomes to treatment with FGFR inhibitors, Loeffler et al. showed that FGFR3 mutation status could be accurately predicted from H&E slides [38]. However, a substantial decrease in AUC was observed during external validation.

Jang et al. addressed the question of how a deep learning model trained using frozen WSIs would perform compared to a model trained using FFPE WSIs [39]. Applied to five clinically actionable genes in colorectal cancer, the authors found that the difference in performance of the model trained on frozen and FFPE WSIs varied across the genes. For example, higher AUCs were achieved for APC and KRAS using frozen WSIs over FFPE WSIs, while frozen WSIs resulted in a poorer performance for SMAD4 than FFPE slides. This result may be attributable to the manner in which tissue specimens of the The Cancer Genome Atlas (TCGA) were collected, since frozen tissue samples are cut directly from the same specimen used for molecular testing, while FFPE samples are cut from a separate tissue specimen. Therefore, frozen WSIs of the TCGA may provide the best representation of the tissue used for molecular testing, despite FFPE methods better preserving cellular morphology and producing less artefacts. Furthermore, the authors showed that a model trained using only TCGA data had poor generalisability to an external data set of South Korean patients. Further studies would be necessary to better understand whether this poor generalisability is attributable to batch effects in sample preparation or ethnic differences between the cohorts.

In addition to applying deep learning to a single cancer type, researchers have shown that a single neural network may be applied across different tissues. Noorbakhsh et al. trained a neural network to predict the mutation status of TP53 using patients with a single cancer type, and showed that the model could generalise to other tissues with comparable results to self-cohort models [44]. For example, a model trained on breast cancer data achieved AUCs of 0.72, 0.71 and 0.67 on lung adenocarcinoma, stomach adenocarcinoma and bladder urothelial carcinoma test sets, respectively, while also achieving similar performance on the lung adenocarcinoma cases to Coudray et al. [34]. However, the authors found a lower performance of the model when applied to colon adenocarcinoma, suggesting that mutated TP53 may confer a different histomorphological alteration in this tissue than in lung, stomach and breast. It is also worth noting that, in the same study, fully training the deep learning model yielded an improvement in AUC of 0.12 in the TCGA’s lung adenocarcinoma cohort when compared with using a transfer learning approach.

Larger-scale, pan-cancer, studies have also attempted to predict a myriad of genomic alterations across cancer types using the TCGA. Kather et al. trained a single ShuffleNet neural network using WSIs from over 5000 patients across 14 major tumour types in the TCGA [43]. It was found that at least one mutated gene could be significantly inferred from WSIs in 13 of the 14 cancer types. The authors also compared a model trained using all mutations to a model where variants used to label samples were restricted to known oncogenic drivers. Restricting variants to putative oncogenic drivers improved model performance in a number of cases, such as predicting EGFR mutation status in lung adenocarcinoma. Comparing the performance of the neural network trained on frozen slides and FFPE slides, the authors found that molecular inference using deep learning generally worked better on frozen slides. Furthermore, the authors report that removing tiles containing non-tumour tissue did not drastically reduce model performance compared with a weakly-supervised approach. In addition to predicting genetic variants, the neural network was applied to predict MSI, consensus molecular subtypes and CpG island methylator phenotype in colorectal cancer as well as hormone receptor status and gene expression signatures in breast cancer.

Published around the same time, Fu et al. showed that a fine-tuned Inception-v3 model trained on on 17,355 H&E-stained frozen WSIs from the TCGA could be used to predict whole-genome duplications (WGDs), chromosome arm gains and losses, focal amplifications and deletions, as well as point mutations in driver genes [42]. WGD status could be predicted for 19 of the 27 cancer types studied. It was also found that tiles predicted as WGD had larger mean nucleus size and intensity than those predicted to be near-diploid. In total, 151 driver gene-cancer pairs were tested for histopathology associations, 43 of which could be accurately predicted. Notably, TP53 mutation status could be predicted in 12 of the 27 cancer types, while BRAF mutation status could be predicted in thyroid tumours with a reported AUC of 0.92. Both Kather et al. and Fu et al.’s results indicate that in certain cases, gnomic alterations can result in the same histomorphological alterations across tissues.

Collecting sufficient data to train such deep learning models in histology comes at significant financial investment and researchers often rely on large consortia such as the TCGA. For this reason, researchers have investigated augmenting relatively small training data sets using a GAN. Using a combined TCGA and internal data set of 266 glioma patients, Liu et al. sought to investigate how GAN-generated images could be used to improve the performance of predicting isocitrate dehydrogenase mutation status [41]. It was found that augmenting the resulting 24,000 tiles with just 3000 GAN-generated images yielded an improvement in AUC of 0.07. However, a decrease in performance was observed as greater numbers of GAN-generated images were included in the training data set and the GAN-augmented data set fell short of the AUC achieved when including all 921 patients in the TCGA cohort.

Researchers have also shown that image-based mutation classification may correlate with gene expression data. In a study which predicted papillary thyroid carcinomas as BRAF or RAS mutated, Tsou et al. found that mRNA-derived expression patterns correlated with the deep learning model’s predictions, enabling the classification of tumours harbouring neither BRAF nor RAS mutations to be classified as BRAF- or RAS-like [40]. The authors propose that driver mutations in BRAF and RAS dictate downstream signalling cascades which is reflected in both the histopathological features and molecular genetics of the tumour.

### 3.2. Predicting Gene Expression and Hormone Receptor Status

Cancers are known to be characterised by massive transcriptional dysregulations resulting from epigenomic modifications. Despite its potential as a prognostic and predictive biomarker in cancer, gene expression is not routinely assayed due to costs associated with transcriptome profiling as well as high technical and sampling variability [45]. On the other hand, hormone receptor status is routinely assayed in breast cancer cases through molecular immunohistochemistry (IHC) and serves as an important biomarker for endocrine therapy response as well as prognosis. For example, progesterone receptor (PR)-positive patients are found to have a better survival rates than PR-negative patients [46], while breast cancers with human epidermal growth factor receptor 2 (HER2) overexpression may be treated with US Food and Drug Administration (FDA)-approved targeted therapy trastuzumab [47]. However, IHC has several limitations such as potential variation in tissue specimen preparation as well as the subjective interpretation of IHC-stained slides by pathologists. Recently, researchers have begun investigating alternative methods of estimating gene expression and assaying hormone receptor status directly from digitised histopathology slides. This section summarises recent literature concerned with estimating gene expression and hormone receptor status in cancer using deep learning applied to histopathology slides. Identified studies concerned with estimating gene expression and hormone receptor status from WSIs using deep learning are summarised in Table 2.

The outcome of measuring gene expression in precision oncology is generally a decision as to whether or not to administer a drug to a patient. Therefore, early efforts to estimate gene expression from WSIs using deep learning applied a binary prediction approach, in which the status of a selected gene was predicted to be over-expressed or under-expressed. For example, Sha et al. applied a multi-field-of-view deep learning model to predict PD-L1 expression status in non-small cell lung cancer [48]. The proposed multi-field-of-view method integrates tiles of different sizes to account for the fact that both large tissue structures and nuclear morphology may be affected by PD-L1 expression. Using a relatively shallow ResNet18 deep learning architecture, an AUC of 0.80 was achieved

BRCA1-associated protein 1 (BAP1) expression is predictive of metastasis in uveal melanoma [49]. One study showed that deep learning could be used to predict BAP1 expression status in H&E-stained uveal melanoma WSIs with near ophthalmic pathologist performance on an internal test cohort [50]. In this study, a DenseNet neural network architecture outperformed three other state-of-the-art neural network architectures, as well as a support vector machine. A later study by Zhang et al. reportedly outperformed these results using a larger data set [51]. Here, a ResNet18 model was used to extract feature vectors from tiles before assembling a set of feature vectors corresponding to a single slide into a feature map according to their spatial locations. A U-Net auto-encoder-decoder was then used to output a probability map on which an element-wise product with the original tumour mask could be applied, resulting in a tumour probability heat map.

In an effort to go beyond binary predictions of single-gene expression, researchers have recently attempted to quantify the expression of genes using H&E-stained WSIs. Schmauch et al. developed a deep learning framework based on ResNet classifications to quantify the expression of over 17,000 genes across 28 tumour types in the TCGA [52]. Although no genes could be significantly predicted in all 28 cancer types, the expression of several genes could be accurately predicted in multiple cancers, indicating that altered gene expression in certain cases confers the same histomorphological changes across tissues. Schmauch et al. also showed that spatial gene expression information for certain genes could be derived from bulk RNA-seq profiles using their methods.

He et al. advanced these results by integrating spatial gene expression data and tumour morphology in breast cancer using a deep learning model termed ST-Net [53]. Reportedly, the methods developed in this study were successful in determining the spatial variation in the expression of 102 genes, including several breast cancer biomarkers such as GNAS, FASN, and DDX5. External validation carried out on the TCGA-BRCA cohort showed that the trained deep learning model could predict bulk expression profiles for 55 genes by averaging ST-Net’s predictions. Furthermore, the authors showed that ST-Net-derived expression profiles had similar performance to real bulk RNA-seq data in distinguishing histological subtypes. In efforts to elucidate the histopathological features which influenced the deep learning model’s predictions, the authors found that enlarged and less-elongated nuclei tended to correlate with higher expression levels.

Levy-Jurgenson et al. trained deep learning models to spatially resolve bulk mRNA and miRNA expression levels in 10 breast and 5 lung cancer traits [54]. Molecular heat maps could then be generated for each trait from which a heterogeneity score could be derived. Notable results include the model’s performance at predicting mi-R-17-5p expression with AUCs of 0.87 and 0.95 in breast and lung samples, respectively. Additionally, the authors found that patients with high derived heterogeneity scores were significantly linked to poor survival in breast cancer, despite no association existing between the molecular traits and the heterogeneity scores.

In a study predicting PAM50 expression-based molecular subtypes from breast cancer WSIs, Jaber et al. were able to show that tumours predicted as heterogeneous had survival intermediate between Luminal A and Basal patients, as well as more varied levels of hormone receptor expression patterns [55].

Naik et al. utilised a combined data set of the TCGA and Australian Breast Cancer Tissue Bank (ABCTB) to train and validate a deep learning model to predict estrogen receptor (ER) status as well as PR status and HER2 status [56]. Following a manual evaluation of the histomorphological patterns which were associated with ER-status, the authors found that low mitotic rate and uniform cells with small nuclei, among other features, were more likely to be associated with tiles predicted as ER-positive. In contrast, tiles predicted to be ER-negative by the deep learning model contained necrotic debris with reactive lymphoid cells and macrophages removing the debris. Meanwhile, low attention weights were assigned by the model to tiles with fat tissue, connective tissue, and tiles with macrophages laden with debris and fat, demonstrating that the model learned that these features are uninformative when predicting ER status.

In a study by Anand et al., HER2 overexpression was predicted from H&E-stained histopathology slides with an AUC of 0.76 on an independent test set from the TCGA [57]. Three separate neural networks were used in the approach described in this study. The first, a pretrained U-Net based nucleus detector, was used to sample tiles with high numbers of nuclei. The second neural network consisted of a custom CNN to classify nuclei as tumour or non-tumour, while the third neural network consisted of a custom HER2 status classifier.

Rawat et al. employed two neural networks for the prediction of ER, PR, and HER2 status from WSIs [58]. Using the ABCTB as an independent test set, AUCs of 0.89, 0.81 and 0.79 were achieved for ER-status, PR-status, and HER2-status, respectively. Interestingly, the authors found that GAN-based stain normalisation almost doubled the accuracy to the deep learning model, highlighting the role that variations in sample preparation may play when generalising deep learning models in digital pathology.

### 3.3. Predicting Tumour Mutational Burden

While no standardised definition exists, TMB is typically defined as the number of non-synonymous mutations per megabase of exome [59]. TMB has been hailed as an emerging surrogate biomarker for immunotherapies due to its relationship with neoantigens [59]. Although only a minority of somatic mutations in tumours give rise to neoantigens, it is hypothesised that tumours with a greater number of somatic mutations will have increased levels of neoantigens [59]. These neoantigens can then be recognised by CD8+ T-cells, resulting in an increased immune response [60]. In 2020, the FDA granted approval for the use of the immunotherapy pembrolizumab to treat patients with unresectable or metastatic TMB-high solid tumours [61]. Currently, whole-exome sequencing (WES) is considered gold-standard for measuring TMB, however, this is primarily confined to a research setting due to the high cost and complexity of associated with WES. For this reason, researchers have begun investigating alternative methods of estimating TMB, such as using gene panels [62] and applying deep learning to histopathology slides [63]. This section summarises recent literature concerned with estimating TMB using deep learning applied to histopathology slides. Identified studies concerned with estimating TMB from WSIs using deep learning are summarised in Table 3.

Due to the high dynamic range of TMB values, it is considered impractical to directly predict TMB values. Instead, cohorts are typically stratified into TMB-low and TMB-high groups based on a defined threshold, thus resulting in a binary classification problem which can be tackled using a neural network. The first published study found to apply these methods was carried out by Wang et al. in the context of gastric and colon cancer [64]. The performance of eight state-of the-art deep learning models was compared on TCGA stomach adenocarcinoma (STAD) and colon adenocarcinoma (COAD) data sets separately. Interestingly, Googlenet was found to have the highest AUC on the STAD data set while VGG19 was the best performing model on the COAD data set, indicating that the optimal choice of neural network architecture may not only be problem-specific, but also specific to the data set at hand. It is worth noting that the difference in performance between certain pairs of models was found to be substantial, for example, on the TCGA-COAD data set, VGG19 achieved an AUC of 0.82, while the Inception-v3 model trained using the same set of hyperparameters achieved an AUC of 0.73. A possible explanation for these discrepancies is that the optimal set hyperparameters may be model-specific. The impact of the selected probability threshold used to separate classes was also investigated, revealing that optimal thresholds differed between the two cancer types. The authors acknowledged that using the upper tertile of the TMB distribution across each data set to label patients as TMB-low or TMB-high may not be optimal.

In a later study by Jain et al., the authors investigated the dependence of model performance on the selected TMB threshold in lung adenocarcinoma [63]. Similar AUCs were achieved for the median, upper tertile of the TMB distribution, and 10 mutations per megabase, while a slight drop in performance was observed when setting the TMB threshold to the upper quartile. In addition to optimising the TMB threshold, the authors sought to elucidate how magnification could affect model performance. It was found that tiles sampled at 20× magnification generally yielded better model performance, however, performance was substantially improved when aggregating the predictions at three magnifications using a random forest classifier. These results may indicate that levels of TMB influence both the spatial arrangement of tissue, observed at low magnification, and cell structure, observed at high magnification. Despite demonstrating that patients can be stratified by TMB from WSIs, the two aforementioned studies report no efforts to elucidate the histomorphological changes identified by deep learning models to make such predictions.

A later study published by Shimada et al. aimed to identify the histopathological characteristics associated with possessing a high TMB, revealing that slides predicted to be TMB-high by a deep learning model applied to a colorectal cancer cohort were enriched for tumour-infiltrating lymphocytes (TILs) [65]. In addition to testing the association between TMB-high predictions and a number of clinicopathological features, the authors found that mismatch repair (MMR) status was also significantly associated with TMB-high, showing that the same model could generalise to predict MSI in patients.

### 3.4. Predicting Microsatellite Instability

MSI arises as a result of deficiencies in MMR pathways, such as mutations in MMR genes and hypermethylation of the MLH1 gene promotor [66]. MMR deficiencies result in the accumulation of mutations in short tandem repeats known as microsatellites [67]. MSI is most prevalent in colorectal cancers, although it is also be observed in number of other cancers. In 2017, the FDA approved the use of anti-PD1 therapy in MSI-high or MMR-deficient solid tumours [68]. Since then, the demand for MSI/MMR testing in the clinic has increased dramatically. At present, polymerase chain reaction and IHC assays are used to test for MSI/MMR in tumours [69]. However, recent research has focused on using deep learning applied to WSIs to identify MSI/MMR cases. MSI/MMR tumours have been found to be characterised by distinct histomorphological features, such as tumour-infiltrating lymphocytes, mucinous differentiation and poor differentiation [70]. This section summarises recent literature concerned with predicting MSI in cancers using deep learning applied to histopathology slides. Identified studies concerned with estimating MSI from WSIs using deep learning are summarised in Table 4.

Several studies were identified which explore the use of deep learning to predict MSI status from histopathology slides, most of which were applied to colorectal cancer. As with predicting TMB, the task is generally approached as binary classification problem, with the aim of stratifying patients according MSI-high or MSI-low (also referred to as microsatellite-stable, or MSS). The earliest published study found to predict MSI from histopathology slides was carried out by Kather et al. in 2019 [71]. Here, a quantitative measure of MSI was derived using tile-level predictions and significant correlations were observed between MSI scores and PD-L1 expression and an interferon-γ signature in colorectal cancer, as well as CD8+ expression signatures in gastric cancer. These results may indicate that different mechanisms of driving MSI exist across cancer types. Additionally, tiles predicted as MSI-high were found to spatially overlap with regions characterised by poor cell differentiation and lymphocyte-rich tumour regions, consistent with previous histopathological knowledge [70].

Kather et al.’s work was used as a benchmark for a number of subsequent studies. Using a distillation framework, which uses a neural network’s previous iteration’s predictions during training in an effort to reduce generalisation errors, Ke et al. achieved improvements in AUC of between 0.01 and 0.04 on the same problems as described in Kather’s 2019 paper [72].

Echle et al. revisited the problem using a large-scale international cohort, MSIDETECT, hailed as the *“first international collaborative effort to validate such a deep learning–based molecular biomarker”* [73]. Interestingly, diminishing performance was observed using training cohorts greater than 5500 patients, at which a maximum AUC of 0.92 was achieved. Echle et al. also demonstrated that while colour normalisation did not result in higher performance on a held-out test set, a slight increase in performance was observed on an external data set, demonstrating that colour normalisation may improve the generalisability of deep learning models in predicting MSI.

Cao et al. developed a MIL-based deep learning model trained on frozen WSIs of TCGA colon adenocarcinoma cases and showed that the model could be generalised to an Asian colorectal cancer cohort of FFPE slides through transfer learning [28]. Fine-tuning the model on just 10% of the Asian colorectal cancer samples improved the model’s AUC by 0.2 compared to generalising the model without transfer learning.

Yamashita et al. compared the performance of an MSI-prediction deep learning model with pathologist’s evaluation and sought to improve the interpretability of the model by assessing ten human-interpretable features known to be associated with MSI [74]. Although MSI is not routinely screened for by pathologists, the author’s model exceeded the performance of pathologists at detecting MSI in colorectal cancer, while also outperforming Kather et al.’s original model. Of ten hand-crafted histomorphological features known to have associations with MSI, only three, the presence of more than two TILs per high-power field, mucinous differentiation, and absence of dirty necrosis, were found to be significantly with MSI status upon multivariable analysis. The same three features were found to be significantly associated with the deep learning model’s predictions, demonstrating a degree of explainability for the model.

The most recently published study showed that frozen tissue specimens resulted in better model performance than FFPE tissues for predicting MSI in colorectal cancer [75]. Additionally, the authors showed that a model trained on colorectal samples was unable to discriminate MSI-high and MSI-low samples in metastasized tumours, demonstrating that features associated with MSI in one type of tissue may not be associated with MSI in another tissue type.

Another recent study sought to investigate how synthetic images could be used to augment data sets for training deep learning models to predict MSI status in colorectal cancer [27]. The authors were able to show that images produced using a GAN retained histomorphological features associated with MSI, such as poor differentiation and intraepithelial lymphocytosis. These synthetic images could then be identified by a CNN. Augmenting a training data set of real images with GAN-generated images yielded an improvement in AUC of 0.035. Despite these promising results, further studies will be necessary to validate the use of GAN-generated images for training deep learning models, particularly applied to other molecular features such as mutated genes or expression.

## 4. Discussion

The digitisation of clinical data over the past decade is enabling a revolution in healthcare through the use of artificial intelligence. Namely, computer vision methods applied to medical imaging data have seen numerous successes both in a research and clinical setting [12]. While histopathology is considered gold-standard in cancer diagnosis, new precision treatments often require the identification of clinically actionable, molecular biomarkers through genomics assays. Since the first published study aiming to predict molecular genetics from histopathology slides in 2018 [34], there has been a growing number of publications applying such methods to a range of informative cancer biomarkers. While using deep learning to infer molecular biomarkers from histopathology slides offers a promising alternative to costly and time-consuming molecular tests, many obstacles remain to be overcome.

As demonstrated through this review of literature, the performance of deep learning models in digital pathology is dependent on a number parameters related to study design, such as the neural network architecture, sample preparation, the size of the cohorts available and the methods used for establishing ground-truth labels for training, among others. For example, Wang et al. found a discrepancy in AUC of almost 0.1 between a VGG19 neural network and an Inception-v3 neural network trained on the same problem [64]. Echle et al. found that diminishing performance was observed after increasing cohort size past 5500 patients when predicting MSI status [73], while Kather et al. found that using different methods of defining mutated genes in training samples yielded different results in certain genes [43].

Notably, the generalisability of these deep learning models to populations of different ethnic groups remains poorly understood. For example, Kather et al. found that their MSI classifier trained on a TCGA data set made up of predominantly Western populations generalised poorly to a Japanese cohort [71]. Although a similar result was obtained by Cao et al., the authors showed that fine-tuning the model on a small subset of a cohort with a different ethnic background vastly improved model generalisability [28]. These studies demonstrate the need for large-scale multi-center research efforts, such as the work carried out by Echle et al. using the MSIDETECT consortium [73], as well as rigorous external validation of results in order to mitigate the risk of bias in clinical practice.

Substantial time and financial investment is required to recruit cohorts of sufficient size to maximise and reliably estimate the performance of deep learning models in digital pathology. Therefore, future studies will likely rely on collaborations between multiple research centres to attain the level of performance and testing required to eventually enable safe and effective clinical decisions to be made based on molecular biomarkers detected through deep learning applied to histopathology slides. Additionally, Krause et al. and Liu et al.’s use of synthetic images to augment digital pathology data sets has been shown to merit further research and such methods may prove invaluable in the future when assembling large, shareable data sets [27,41].

Furthermore, the importance of model interpretability in digital pathology studies cannot be overstated if such methods are to be effectively integrated into clinical workflows. Without thorough assessments of the features leading to a model’s decision, it is impossible to ensure that a deep learning model is not exploiting artefacts in data sets to obtain predictions. Additionally, “black-box” models make it difficult for medical professionals to trust that predictions can effectively complement other measurable data in making clinical decisions. While many recently published studies make efforts to explain models’ predictions using human-interpretable features, such practice should continue to be encouraged among researchers in order to facilitate the translation of novel diagnostic strategies based on deep learning to the clinic.

## Figures and Tables

**Table 1:** Comparison of studies aimed at predicting mutations from digitised histolpathology slides using deep learning.

Reference	Description	Cohorts	Num. of Samples (ext. Validation)	External Validation	Neural Network	Outcome Measures
Coudray, 2018 [34]	Predicted the mutation status of 6 of 10 selected genes in lung cancer.	TCGA-LUAD	567 (1)	Yes	Inception-v3	AUC range 0.73 (KRAS) to 0.85 (STK11)
Tsou, 2019 [40]	Predicted BRAF V600E or RAS mutant in papillary thyroid carcinoma.	TCGA-PTC	103	No	Inception-v3	AUC 0.951
Liu, 2020 [41]	GAN-based data augmentation to improve prediction of IDH mutation status in glioma.	TCGA-GBM, -LGG, internal data set	266	No	ResNet50	AUC 0.927
Chen, 2020 [35]	Predicted the mutation status of 4 of 10 selected genes in liver cancer.	TCGA-HCC, internal data set	387 (101)	Yes	Inception-v3	AUC range 0.71 (ZFX4) to 0.89 (CTNNB1)
Fu, 2020 [42]	Predicted a number of mutations across 28 cancers.	TCGA, METABRIC, BASIS	17,355 (622)	Yes	Inception-v4	AUC 0.73 (WGD)
Kather, 2020 [43]	Predicted mutations and gene expression signatures across 14 cancers.	TCGA, DACHS	>5000 (408)	Yes	ShuffleNet	AUC 0.77 (BRAF in CRC); AUC 0.66 (CIMP-high in CRC)
Jang, 2020 [39]	Predicted the mutation status of 5 genes in colorectal cancer.	TCGA-READ, -COAD, internal data set	629 (142)	Yes	Inception-v3	AUC range 0.693 (SMAD4) to 0.809 (TP53) on frozen; AUC range 0.645 (KRAS) to 0.783 (TP53) on FFPE
Noorbakhsh, 2020 [44]	Predicted TP53 mutation status across cancer types.	TCGA-BRCA, -LUAD, -STAD, -COAD, -BLCA	27,815 (2115)	Yes	Inception-v3	AUC range 0.65 to 0.80
Wu, 2020 [37]	Predicted BRAF V600E status in papillary thyroid carcinoma.	Internal data set, PCam-BRCA, PAIP-LIHC	439	Yes	DenseNet-121	AUC 0.884
Yang, 2021 [36]	Predicted mutation status of immune-related genes in lung cancer.	TCGA-LUAD, -LUSC	180	No	ResNet	AUC range 0.71 to 0.87
Loeffler, 2021 [38]	Predicted mutation status of FGFR3 in bladder cancer.	TCGA-BLCA, Aachen cohort	327 (182)	Yes	ShuffleNet	AUC 0.70 (TCGA-BLCA); AUC 0.62 (ext. validation)

**Table 2:** Comparison of studies aimed at predicting gene expression/hormone receptor status from digitised histopathology slides using deep learning.

Reference	Description	Cohorts	Num. of Samples (ext. Validation)	External Validation	Neural Network	Outcome Measures
Sha, 2019 [48]	Predicted PD-L1 expression status in lung cancer.	Internal data set	130	No	ResNet18	AUC 0.80
Sun, 2019 [50]	Predicted BAP1 expression status in uveal melanoma.	Internal data sets	17 (30)	Yes	DenseNet-121	AUC-positive 0.99; AUC-negative 0.98
Jaber, 2020 [55]	Predicted PAM50 gene expression- based subtypes in breast cancer.	TCGA-BRCA	1142	No	Inception-v3	AUC 0.82 (Basal/non-Basal)
Rawat, 2020 [58]	Predicted hormone receptor status in breast cancer.	TCGA-BRCA, ABCTB	939 (2351)	Yes	ResNet34	AUC 0.89 (ER); AUC 0.81 (PR); AUC 0.79 (HER2)
He, 2020 [53]	Predicted spatial gene expression profiles in breast cancer.	Internal data set, 10x Genomics BC, TCGA-BRCA	23 (1094)	Yes	DenseNet-121	Pearson’s R range for top 5 genes 0.43–0.54
Anand, 2020 [57]	Predicted HER2 overexpression in breast cancer.	Warwick, TCGA-BRCA	52 (45)	Yes	U-Net and custom architecture	AUC 0.76
Schmauch, 2020 [52]	Predicted gene expression of multiple genes across cancers.	TCGA	8725 (369)	Yes	ResNet50	Pearson’s R 0.47 (MKI67 in LIHC), 0.43 (CD3D in COAD)
Zhang, 2020 [51]	Predicted BAP1 expression status in uveal melanoma.	Internal data set	184	No	ResNet18	AUC 0.93
Levy-Jurgenson, 2020 [54]	Predicted spatially resolved transctiptomics from bulk mRNA and miRNA expression.	TCGA-BRCA, -LUAD	761 BRCA, 469 LUAD	No	Inception-v3	AUC 0.95 (miR-17)
Naik, 2020 [56]	Predicted hormone receptor status in breast cancer.	ABCTB, TCGA-BRCA	2535 ABCTB, 1014 BRCA	Yes	ResNet50	AUC 0.92

**Table 3:** Comparison of studies aimed at predicting tumour mutational burden from digitised histopathology slides using deep learning.

Reference	Description	Cohorts	Num. of Samples (ext. Validation)	External Validation	Neural Network	Outcome Measures
Jain, 2020 [63]	Predicted TMB in lung adenocarcinoma.	TCGA-LUAD	760	No	Inception-v3	AUPRC 0.92
Wang, 2020 [64]	Predicted TMB in gastrointestinal cancer.	TCGA-STAD, -COAD, -READ	644	No	Resnet50, Googlenet, Inception v3, Alexnet, VGG19, Squeezenet, Densenet	AUC 0.75 STAD (Googlenet); AUC 0.82 COAD (VGG19)
Shimada, 2021 [65]	Predicted TMB in colorectal cancer.	TCGA-CRC, Japanese-CRC	278	No	Inception-v3	AUPRC 0.91

**Table 4:** Comparison of studies aimed at predicting microsatellite instability from digitised histopathology slides using deep learning.

Reference	Description	Cohorts	Num. of Samples (ext. Validation)	External Validation	Neural Network	Outcome Measures
Kather, 2019 [71]	Predicted MSI status in gastointestinal cancer.	TCGA-STAD, -CRC, DACHS	315 STAD, 747 CRC, (378)	Yes	ResNet18	AUC 0.81 TCGA-STAD; AUC 0.84 TCGA-CRC-KR; AUC 0.77 TCGA-CRC-DX
Ke, 2019 [72]	Predicted MSI status in colorectal cancer.	TCGA-CRC	387 CRC-KR, 360 CRC-DX	No	AlexNet, ResNet, VGG, Inception-v3	Accuracy 98.8% (AlexNet)
Cao, 2020 [28]	Predicted MSI status in colorectal cancer.	TCCG-COAD, internal data set	429 COAD, 785 internal	Yes	ResNet18	AUC 0.88 (TCGA-COAD); AUC 0.85 (internal)
Echle, 2020 [73]	Predicted MSI status in colorectal cancer using a large, multicenter cohort.	MSIDETECT	6406 (771)	Yes	ShuffleNet	AUC 0.96
Yamashita, 2021 [74]	Predicted MSI status in colorectal cancer.	Internal data set, TCGA-CRC	100 (484)	Yes	MobileNetV2	AUC 0.779
Krause, 2021 [27]	Used GAN-generated images to improve prediction of MSI in colorectal cancer.	TCGA-CRC, NLCS	256 CRC, 1457 NLCS	No	Conditional GAN, ShuffleNet	AUC 0.742 (TCGA-CRC); AUC 0.757 (NLCS); AUC 0.777 (real and synthetic data)
Lee, 2021 [75]	Predicted MSI status in colorectal cancer.	TCGA-CRC, SMH	1920 (365)	Yes	Inception-v3	AUC 0.972 (TCGA-CRC); AUC 0.787 (SMH)

## Abbreviations

The following abbreviations are used in this manuscript:

EGFR	Epidermal growth factor receptor
NSCLC	Non-small cell lung cancer
TMB	Tumour mutational burden
MSI	Microsatellite instability
MIL	Multiple-instance learning
CNN	Convolutional neural network
WSI	Whole-slide image
FFPE	Formalin-fixed paraffin-embedded
H&E	Hematoxylin and eosin
AUC	Area under the ROC curve
TCGA	The Cancer Genome Atlas
WGD	Whole-genome duplication
GAN	Generative adversarial network
IHC	Immunohistochemistry
PR	Progesterone receptor
HER2	Human epidermal growth factor receptor 2
FDA	US Food and Drug Administration
BAP1	BRCA1-associated protein 1
ABCTB	Australian Breast Cancer Tissue Bank
ER	Estrogen rceptor
WES	Whole-exome sequencing
STAD	Atomach adenomcarcinoma
COAD	Colon adenocarcinoma
TIL	Tumour-infiltrating lymphocytes
MMR	Mismatch repair

## References

[1] Sequist L.V., Lynch T.J. (2008). EGFR Tyrosine Kinase Inhibitors in Lung Cancer: An Evolving Story. Annu. Rev. Med..

[2] Bollag G., Hirth P., Tsai J., Zhang J., Ibrahim P.N., Cho H., Spevak W., Zhang C., Zhang Y., Habets G. (2010). Clinical efficacy of a RAF inhibitor needs broad target blockade in BRAF-mutant melanoma. Nature.

[3] Tsao M.S., Aviel-Ronen S., Ding K., Lau D., Liu N., Sakurada A., Whitehead M., Zhu C.Q., Livingston R., Johnson D.H. (2007). Prognostic and predictive importance of p53 and RAS for adjuvant chemotherapy in non-small-cell lung cancer. J. Clin. Oncol..

[4] Davis A.A., Patel V.G. (2019). The role of PD-L1 expression as a predictive biomarker: An analysis of all US food and drug administration (FDA) approvals of immune checkpoint inhibitors. J. Immunother. Cancer.

[5] Kim H., Chung J.H. (2019). PD-L1 Testing in Non-Small Cell Lung Cancer: Past, Present, and Future. J. Pathol. Transl. Med..

[6] Hellmann M.D., Ciuleanu T.E., Pluzanski A., Lee J.S., Otterson G.A., Audigier-Valette C., Minenza E., Linardou H., Burgers S., Salman P. (2018). Nivolumab plus Ipilimumab in Lung Cancer with a High Tumor Mutational Burden. N. Engl. J. Med..

[7] Zhao P., Li L., Jiang X., Li Q. (2019). Mismatch repair deficiency/microsatellite instability-high as a predictor for anti-PD-1/PD-L1 immunotherapy efficacy. J. Hematol. Oncol..

[8] Viros A., Fridlyand J., Bauer J., Lasithiotakis K., Garbe C., Pinkel D., Bastian B.C. (2008). Improving melanoma classification by integrating genetic and morphologic features. PLoS Med..

[9] Ninomiya H., Hiramatsu M., Inamura K., Nomura K., Okui M., Miyoshi T., Okumura S., Satoh Y., Nakagawa K., Nishio M. (2009). Correlation between morphology and EGFR mutations in lung adenocarcinomas. Significance of the micropapillary pattern and the hobnail cell type. Lung Cancer.

[10] Brockmoeller S., Young C., Lee J., Arends M.J., Wilkins B.S., Thomas G.J., Oien K.A., Jones L., Hunter K.D. (2019). Survey of UK histopathology consultants’ attitudes towards academic and molecular pathology. J. Clin. Pathol..

[11] Märkl B., Füzesi L., Huss R., Bauer S., Schaller T. (2021). Number of pathologists in Germany: Comparison with European countries, USA, and Canada. Virchows Arch..

[12] Benjamens S., Dhunnoo P., Meskó B. (2020). The state of artificial intelligence-based FDA-approved medical devices and algorithms: An online database. NPJ Digit. Med..

[13] Deng J., Dong W., Socher R., Li L.J., Li K., Li F.-F. ImageNet: A Large-Scale Hierarchical Image Database. Proceedings of the 2009 IEEE Conference on Computer Vision and Pattern Recognition.

[14] LeCun Y.A., Bottou L., Orr G.B., Müller K.R. (2012). Efficient BackProp. Lecture Notes in Computer Science (Including Subseries Lecture Notes in Artificial Intelligence and Lecture Notes in Bioinformatics).

[15] Bishop C.M. (2006). Pattern Recognition and Machine Learning.

[16] LeCun Y., Bengio Y. (1995). Convolutional networks for images, speech, and time series. The Handbook of Brain Theory and Neural Networks.

[17] Arvaniti E., Fricker K.S., Moret M., Rupp N., Hermanns T., Fankhauser C., Wey N., Wild P.J., Rüschoff J.H., Claassen M. (2018). Automated Gleason grading of prostate cancer tissue microarrays via deep learning. Sci. Rep..

[18] Kather J.N., Krisam J., Charoentong P., Luedde T., Herpel E., Weis C.A., Gaiser T., Marx A., Valous N.A., Ferber D. (2019). Predicting survival from colorectal cancer histology slides using deep learning: A retrospective multicenter study. PLoS Med..

[19] Gao X.H., Li J., Gong H.F., Yu G.Y., Liu P., Hao L.Q., Liu L.J., Bai C.G., Zhang W. (2020). Comparison of Fresh Frozen Tissue With Formalin-Fixed Paraffin-Embedded Tissue for Mutation Analysis Using a Multi-Gene Panel in Patients With Colorectal Cancer. Front. Oncol..

[20] Khened M., Kori A., Rajkumar H., Krishnamurthi G., Srinivasan B. (2021). A generalized deep learning framework for whole-slide image segmentation and analysis. Sci. Rep..

[21] Priego-Torres B.M., Sanchez-Morillo D., Fernandez-Granero M.A., Garcia-Rojo M. (2020). Automatic segmentation of whole-slide H&E stained breast histopathology images using a deep convolutional neural network architecture. Expert Syst. Appl..

[22] Castiglioni I., Rundo L., Codari M., Di Leo G., Salvatore C., Interlenghi M., Gallivanone F., Cozzi A., D’Amico N.C., Sardanelli F. (2021). AI applications to medical images: From machine learning to deep learning. Phys. Med..

[23] Howard F.M., Dolezal J., Kochanny S., Schulte J., Chen H., Heij L., Huo D., Nanda R., Olopade O.I., Kather J.N. (2020). The Impact of Digital Histopathology Batch Effect on Deep Learning Model Accuracy and Bias. bioRxiv.

[24] Tellez D., Litjens G., Bándi P., Bulten W., Bokhorst J.M., Ciompi F., van der Laak J. (2019). Quantifying the effects of data augmentation and stain color normalization in convolutional neural networks for computational pathology. Med. Image Anal..

[25] Russakovsky O., Deng J., Su H., Krause J., Satheesh S., Ma S., Huang Z., Karpathy A., Khosla A., Bernstein M. (2014). ImageNet Large Scale Visual Recognition Challenge. Int. J. Comput. Vis..

[26] Tan C., Sun F., Kong T., Zhang W., Yang C., Liu C. (2018). A Survey on Deep Transfer Learning. Proceedings of the 27th International Conference on Artificial Neural Networks.

[27] Krause J., Grabsch H.I., Kloor M., Jendrusch M., Echle A., Buelow R.D., Boor P., Luedde T., Brinker T.J., Trautwein C. (2021). Deep learning detects genetic alterations in cancer histology generated by adversarial networks. J. Pathol..

[28] Cao R., Yang F., Ma S.C., Liu L., Zhao Y., Li Y., Wu D.H., Wang T., Lu W.J., Cai W.J. (2020). Development and interpretation of a pathomics-based model for the prediction of microsatellite instability in Colorectal Cancer. Theranostics.

[29] Zhou B., Khosla A., Lapedriza A., Oliva A., Torralba A. (2016). Learning Deep Features for Discriminative Localization. Proceedings of the IEEE Computer Society Conference on Computer Vision and Pattern Recognition.

[30] Bach S., Binder A., Montavon G., Klauschen F., Müller K.R., Samek W. (2015). On pixel-wise explanations for non-linear classifier decisions by layer-wise relevance propagation. PLoS ONE.

[31] Simonyan K., Vedaldi A., Zisserman A. Deep Inside Convolutional Networks: Visualising Image Classification Models and Saliency Maps. Proceedings of the 2nd International Conference on Learning Representations, ICLR 2014—Workshop Track Proceedings.

[32] Hodis E., Watson I.R., Kryukov G.V., Arold S.T., Imielinski M., Theurillat J.P., Nickerson E., Auclair D., Li L., Place C. (2012). A landscape of driver mutations in melanoma. Cell.

[33] Kumari N., Singh S., Haloi D., Mishra S.K., Krishnani N., Nath A., Neyaz Z. (2019). Epidermal Growth Factor Receptor Mutation Frequency in Squamous Cell Carcinoma and Its Diagnostic Performance in Cytological Samples: A Molecular and Immunohistochemical Study. World J. Oncol..

[34] Coudray N., Ocampo P.S., Sakellaropoulos T., Narula N., Snuderl M., Fenyö D., Moreira A.L., Razavian N., Tsirigos A. (2018). Classification and mutation prediction from non–small cell lung cancer histopathology images using deep learning. Nat. Med..

[35] Chen M., Zhang B., Topatana W., Cao J., Zhu H., Juengpanich S., Mao Q., Yu H., Cai X. (2020). Classification and mutation prediction based on histopathology H&E images in liver cancer using deep learning. NPJ Precis. Oncol..

[36] Yang Y., Yang J., Liang Y., Liao B., Zhu W., Mo X., Huang K. (2021). Identification and Validation of Efficacy of Immunological Therapy for Lung Cancer From Histopathological Images Based on Deep Learning. Front. Genet..

[37] Wu Z., Huang X., Huang S., Ding X., Wang L. (2020). Direct Prediction of BRAFV600E Mutation from Histopathological Images in Papillary Thyroid Carcinoma with a Deep Learning Workflow. Proceedings of the 4th International Conference on Computer Science and Artificial Intelligence.

[38] Loeffler C.M.L., Ortiz Bruechle N., Jung M., Seillier L., Rose M., Laleh N.G., Knuechel R., Brinker T.J., Trautwein C., Gaisa N.T. (2021). Artificial Intelligence–based Detection of FGFR3 Mutational Status Directly from Routine Histology in Bladder Cancer: A Possible Preselection for Molecular Testing?. Eur. Urol. Focus.

[39] Jang H.J., Lee A., Kang J., Song I.H., Lee S.H. (2020). Prediction of clinically actionable genetic alterations from colorectal cancer histopathology images using deep learning. World J. Gastroenterol..

[40] Tsou P., Wu C.J. (2019). Mapping Driver Mutations to Histopathological Subtypes in Papillary Thyroid Carcinoma: Applying a Deep Convolutional Neural Network. J. Clin. Med..

[41] Liu S., Shah Z., Sav A., Russo C., Berkovsky S., Qian Y., Coiera E., Di Ieva A. (2020). Isocitrate dehydrogenase (IDH) status prediction in histopathology images of gliomas using deep learning. Sci. Rep..

[42] Fu Y., Jung A.W., Torne R.V., Gonzalez S., Vöhringer H., Shmatko A., Yates L.R., Jimenez-Linan M., Moore L., Gerstung M. (2020). Pan-cancer computational histopathology reveals mutations, tumor composition and prognosis. Nat. Cancer.

[43] Kather J.N., Heij L.R., Grabsch H.I., Loeffler C., Echle A., Muti H.S., Krause J., Niehues J.M., Sommer K.A., Bankhead P. (2020). Pan-cancer image-based detection of clinically actionable genetic alterations. Nat. Cancer.

[44] Noorbakhsh J., Farahmand S., Foroughi Pour A., Namburi S., Caruana D., Rimm D., Soltanieh-ha M., Zarringhalam K., Chuang J.H. (2020). Deep learning-based cross-classifications reveal conserved spatial behaviors within tumor histological images. Nat. Commun..

[45] McIntyre L.M., Lopiano K.K., Morse A.M., Amin V., Oberg A.L., Young L.J., Nuzhdin S.V. (2011). RNA-seq: Technical variability and sampling. BMC Genom..

[46] Dowsett M., Houghton J., Iden C., Salter J., Farndon J., A’Hern R., Sainsbury R., Baum M. (2006). Benefit from adjuvant tamoxifen therapy in primary breast cancer patients according oestrogen receptor, progesterone receptor, EGF receptor and HER2 status. Ann. Oncol..

[47] Hudis C.A. (2007). Trastuzumab—Mechanism of Action and Use in Clinical Practice. N. Engl. J. Med..

[48] Sha L., Osinski B., Ho I., Tan T., Willis C., Weiss H., Beaubier N., Mahon B., Taxter T., Yip S. (2019). Multi-field-of-view deep learning model predicts nonsmall cell lung cancer programmed death-ligand 1 status from whole-slide hematoxylin and eosin images. J. Pathol. Inform..

[49] Griewank K.G., Van De Nes J., Schilling B., Moll I., Sucker A., Kakavand H., Haydu L.E., Asher M., Zimmer L., Hillen U. (2014). Genetic and clinico-pathologic analysis of metastatic uveal melanoma. Mod. Pathol..

[50] Sun M., Zhou W., Qi X., Zhang G., Girnita L., Seregard S., Grossniklaus H.E., Yao Z., Zhou X., Stålhammar G. (2019). Prediction of BAP1 expression in uveal melanoma using densely-connected deep classification networks. Cancers.

[51] Zhang H., Kalirai H., Acha-Sagredo A., Yang X., Zheng Y., Coupland S.E. (2020). Piloting a deep learning model for predicting nuclear BAP1 immunohistochemical expression of uveal melanoma from hematoxylin-and-eosin sections. Transl. Vis. Sci. Technol..

[52] Schmauch B., Romagnoni A., Pronier E., Saillard C., Maillé P., Calderaro J., Kamoun A., Sefta M., Toldo S., Zaslavskiy M. (2020). A deep learning model to predict RNA-Seq expression of tumours from whole slide images. Nat. Commun..

[53] He B., Bergenstråhle L., Stenbeck L., Abid A., Andersson A., Borg Å., Maaskola J., Lundeberg J., Zou J. (2020). Integrating spatial gene expression and breast tumour morphology via deep learning. Nat. Biomed. Eng..

[54] Levy-Jurgenson A., Tekpli X., Kristensen V.N., Yakhini Z. (2020). Spatial transcriptomics inferred from pathology whole-slide images links tumor heterogeneity to survival in breast and lung cancer. Sci. Rep..

[55] Jaber M.I., Song B., Taylor C., Vaske C.J., Benz S.C., Rabizadeh S., Soon-Shiong P., Szeto C.W. (2020). A deep learning image-based intrinsic molecular subtype classifier of breast tumors reveals tumor heterogeneity that may affect survival. Breast Cancer Res..

[56] Naik N., Madani A., Esteva A., Keskar N.S., Press M.F., Ruderman D., Agus D.B., Socher R. (2020). Deep learning-enabled breast cancer hormonal receptor status determination from base-level H&E stains. Nat. Commun..

[57] Anand D., Kurian N., Dhage S., Kumar N., Rane S., Gann P., Sethi A. (2020). Deep learning to estimate human epidermal growth factor receptor 2 status from hematoxylin and eosin-stained breast tissue images. J. Pathol. Inform..

[58] Rawat R.R., Ortega I., Roy P., Sha F., Shibata D., Ruderman D., Agus D.B. (2020). Deep learned tissue “fingerprints” classify breast cancers by ER/PR/Her2 status from H&E images. Sci. Rep..

[59] Chan T.A., Yarchoan M., Jaffee E., Swanton C., Quezada S.A., Stenzinger A., Peters S. (2019). Development of tumor mutation burden as an immunotherapy biomarker: Utility for the oncology clinic. Ann. Oncol..

[60] Yarchoan M., Johnson B.A., Lutz E.R., Laheru D.A., Jaffee E.M. (2017). Targeting neoantigens to augment antitumour immunity. Nat. Rev. Cancer.

[61] Bersanelli M. (2020). Tumour mutational burden as a driver for treatment choice in resistant tumours (and beyond). Lancet Oncol..

[62] Tang Y., Li Y., Wang W., Lizaso A., Hou T., Jiang L., Huang M. (2020). Tumor mutation burden derived from small next generation sequencing targeted gene panel as an initial screening method. Transl. Lung Cancer Res..

[63] Jain M.S., Massoud T.F. (2020). Predicting tumour mutational burden from histopathological images using multiscale deep learning. Nat. Mach. Intell..

[64] Wang L., Jiao Y., Qiao Y., Zeng N., Yu R. (2020). A novel approach combined transfer learning and deep learning to predict TMB from histology image. Pattern Recognit. Lett..

[65] Shimada Y., Okuda S., Watanabe Y., Tajima Y., Nagahashi M., Ichikawa H., Nakano M., Sakata J., Takii Y., Kawasaki T. (2021). Histopathological characteristics and artificial intelligence for predicting tumor mutational burden-high colorectal cancer. J. Gastroenterol..

[66] Kawakami H., Zaanan A., Sinicrope F.A. (2015). Microsatellite Instability Testing and Its Role in the Management of Colorectal Cancer. Curr. Treat. Opt. Oncol..

[67] Boland C.R., Goel A. (2010). Microsatellite Instability in Colorectal Cancer. Gastroenterology.

[68] Lemery S., Keegan P., Pazdur R. (2017). First FDA Approval Agnostic of Cancer Site—When a Biomarker Defines the Indication. N. Engl. J. Med..

[69] Kather J.N., Halama N., Jaeger D. (2018). Genomics and emerging biomarkers for immunotherapy of colorectal cancer. Semin. Cancer Biol..

[70] De Smedt L., Lemahieu J., Palmans S., Govaere O., Tousseyn T., Van Cutsem E., Prenen H., Tejpar S., Spaepen M., Matthijs G. (2015). Microsatellite instable vs stable colon carcinomas: Analysis of tumour heterogeneity, inflammation and angiogenesis. Br. J. Cancer.

[71] Kather J.N., Pearson A.T., Halama N., Jäger D., Krause J., Loosen S.H., Marx A., Boor P., Tacke F., Neumann U.P. (2019). Deep learning can predict microsatellite instability directly from histology in gastrointestinal cancer. Nat. Med..

[72] Ke J., Shen Y., Guo Y., Wright J.D., Liang X. (2020). A Prediction Model of Microsatellite Status from Histology Images. Proceedings of the ICBET 2020: 2020 10th International Conference on Biomedical Engineering and Technology.

[73] Echle A., Grabsch H.I., Quirke P., van den Brandt P.A., West N.P., Hutchins G.G., Heij L.R., Tan X., Richman S.D., Krause J. (2020). Clinical-Grade Detection of Microsatellite Instability in Colorectal Tumors by Deep Learning. Gastroenterology.

[74] Yamashita R., Long J., Longacre T., Peng L., Berry G., Martin B., Higgins J., Rubin D.L., Shen J. (2021). Deep learning model for the prediction of microsatellite instability in colorectal cancer: A diagnostic study. Lancet Oncol..

[75] Lee S.H., Song I.H., Jang H. (2021). Feasibility of deep learning-based fully automated classification of microsatellite instability in tissue slides of colorectal cancer. Int. J. Cancer.

